# Facets of Spirituality Diminish the Positive Relationship between Insecure Attachment and Mood Pathology in Young Adults

**DOI:** 10.1371/journal.pone.0158069

**Published:** 2016-06-23

**Authors:** Michaela Hiebler-Ragger, Johanna Falthansl-Scheinecker, Gerhard Birnhuber, Andreas Fink, Human Friedrich Unterrainer

**Affiliations:** 1 University Clinic of Psychiatry, Medical University Graz, Graz, Austria; 2 Center for Integrative Addiction Research (Grüner Kreis Society), Vienna, Austria; 3 Institute of Psychology, University of Graz, Graz, Austria; University of Perugia, ITALY

## Abstract

Traditionally, in attachment theory, secure attachment has been linked to parameters of mental health, while insecure attachment has been associated with parameters of psychopathology. Furthermore, spirituality and attachment to God have been discussed as corresponding to, or compensating for, primary attachment experiences. Accordingly, they may contribute to mental health or to mental illness. In this cross-sectional observational study, we investigate attachment styles (Avoidant and Anxious Attachment; ECR-RD), spirituality (Religious and Existential Well-Being; MI-RSWB), and mood pathology (Anxiety, Depression, Somatization; BSI-18) in 481 (76% female) young adults (age range: 18–30 years) who had a Roman Catholic upbringing. In accordance with previous research, we found insecure attachment to be associated with low levels of spirituality. Furthermore, insecure attachment and low levels of spirituality were associated with higher levels of mood pathology. In hierarchical regression analyses, only Anxious Attachment positively predicted all three dimensions of mood pathology while Existential Well-Being–but not Religious Well-Being–was an additional negative predictor for Depression. Our results underline that spirituality can correspond to the attachment style, or may also compensate for insecure attachment. Higher Existential Well-Being–comprised of facets such as hope for a better future, forgiveness and the experience of sense and meaning–seems to have an especially corrective effect on mood pathology, independent of attachment styles. Our findings emphasize the vital role of existential well-being in young adults’ affective functioning, which might be considered in prevention and treatment. Further research in clinical surroundings is recommended.

## Introduction

In attachment theory, attachment is seen as a fundamental psychological and biological drive with evolutionary importance and far-reaching, complex consequences [1]. Formed by early family environment, our attachment system is continually active throughout the lifespan [2]. It adaptively regulates psychological and physiological development [3] and influences day-to-day person/environment transactions [4]. Ideally, it offers a ‘secure base’ from which a person can explore the world as well as a ‘safe haven’ to retreat to when frightened or stressed [5]. While attachment research has already contributed immensely to our understanding of our social nature, ‘an important enterprise for the future is to consider how attachment is differentiated from, and integrated with, other features of development’ (p. 25) [6].

### Attachment styles and mood pathology

In line with Mikulincer and Shaver [7], two basic attachment dimensions can be differentiated: ‘Anxious Attachment’ (AX) and ‘Avoidant Attachment’ (AV). While attachment-secure individuals (low AX and low AV) are able to deal with stressful experiences by relying upon mental representations of previously received support or by actively seeking support in the present [8], insecure individuals react by either hyperactivating (AX) or deactivating (AV) their ‘attachment behavioral system’ [5]. In response to an inconsistent caregiver [9] anxious individuals develop hyperactivating strategies that reflect a compromise between the anger toward unavailable attachment figures and the intense need to be close to them [10]. These strategies involve demanding care [11], worry, and rumination [12]. Accordingly, they often amplify distress and fail to regulate emotions [7]. Following an appraisal that proximity seeking behaviors are unfruitful [13], avoidant individuals develop deactivating strategies that imply distrust in others and a desire to maintain self-reliance [14]. These strategies reflect a compromise between a lack of visible negative emotions and high levels of unconscious unresolved distress [15]. Hyperactivating and deactivating strategies link insecure attachment to elevated rates of disease through three mechanisms [16]: Firstly, insecurely attached individuals have an increased susceptibility and a more extreme physiological response to stress. Secondly, they mostly use external methods (e.g., substance use) to regulate affect and thirdly, they have less effective help-seeking behavior, including an underuse of social support. Accordingly, studies have linked family adversity to a selective attention to negative stimuli in youth [17] and to depressive symptoms among young and middle-aged adults [18]. On the other hand, secure attachment seems to have a protective effect against different risk beliefs and problematic behaviors [19].

### Attachment styles and spirituality

The relationship between believers and God, or other divine figures, often meets the established criteria of attachment relationships and therefore confers the same sorts of psychological advantages [20]. The association between attachment styles and spirituality can be explained by two hypotheses [20]: On the one hand, Bowlby’s [21] correspondence hypothesis that mental models generalize across different attachment relationships may extend to the beliefs about, and the perceived relationships with, God. On the other hand, based on Ainsworth’s [22] compensation hypothesis, an attachment to God may be developed by insecurely attached individuals as a surrogate for positive human attachment figures. Consequently, correspondence and compensation hypotheses can be seen as two pathways to and modes of being religious [23]. With the gradual replacement of early attachment figures by a romantic partner or friend [7], adolescence is a period of turbulence and uncertainty that coincides with ‘the age of religious awakening’ [24], characterized by an increased probability of religious conversion as well as of apostasy. In general, securely attached individuals experience only minor fluctuations in their spiritual belief system while insecurely attached individuals are likely to experience major fluctuations including, but not limited to, sudden conversions [25]. These fluctuations seem to correspond with distressing life events that point to the need for emotional support [26].

The sense of having a secure attachment to God can be linked to the depth of spiritual well-being [27]. This linkage leads to a concept of spirituality as an ‘ability to experience and integrate meaning and purpose in existence through a connectedness with self, others or a power greater than oneself’ (p. 117) [28]. In line with previous work [29], this concept comprises an immanent kind of Existential Well-Being (EWB) and a transcendent kind of Religious Well-being (RWB) [30]. While RWB primarily relates to the relationship with God, EWB comprises life satisfaction and a conviction that life is meaningful but does not specifically refer to any higher power [31,32].

### The present study

The present study examines possible influences of spirituality and attachment style on mood pathology in young adults. Based on previous research [19,33] we hypothesized that insecure attachment would be related to higher levels of mood pathology while higher EWB and RWB would be related to lower levels of mood pathology. Furthermore, considering the correspondence pathway [24], spirituality develops from generalized mental attachment models and a general adoption of a primary caregiver’s values. Accordingly, we hypothesized that a more secure attachment would be related to higher EWB and RWB. Considering the compensation pathway [24], spirituality develops through a controlled effort of regulating distress that runs counter to the mental attachment models developed in early life. Therefore, we also hypothesized that spirituality would have a diminishing effect on mood pathology even after attachment styles have been controlled for.

## Material and Methods

### Sample description and procedure

The sample consisted of 481 participants drawn from a larger research project (*n* = 844) that investigated various personality parameters in students and post-graduates at the University of Graz, Austria. All questionnaires were completed online on a Lime Survey® platform. Participants were selected for our study if they had completed all relevant questionnaires, were students and/or held a university degree, and were between 18 and 30 years of age. As some studies indicate that religious orientations differ in their association with mood pathology [33,34], we focused on the most common religious orientation in Austria and therefore included only participants with a Roman Catholic upbringing. This decision also relied on research indicating differences in the attachment to God between Catholics and the worldwide normal distribution [35].

The study was carried out in accordance with the Declaration of Helsinki. Ethical approval was granted by the Ethics Committee of the University of Graz, Austria.

### Psychometric assessment

#### Attachment

The *Experience in Close Relationships—Revised* (ECR-RD) [36] assesses ‘Anxious Attachment’ (AX) and ‘Avoidant Attachment’ (AV) in adults (18 items per scale). It employs a 7-point likert scale (1 = ‘absolutely disagree’ to 7 = ‘absolutely agree’). Cronbach’s α was .91 for AX and .92 for AV [36].

#### Spirituality

The *Multidimensional Inventory for Religious/Spiritual Well-bein*g (MI-RSWB) [37] contains 48 items which form 6 subscales (8 items per scale). These subscales, ‘Hope Immanent’, ‘Forgiveness’ and ‘Experience of Sense and Meaning’, are components of a total Existential Well-Being (EWB) score, while ‘Hope Transcendent’, ‘General Religiosity’ and ‘Connectedness’ reflect a total Religious Well-Being (RWB) score. Each item is rated on a 6-point likert scale ranging from 1 (‘strongly disagree’) to 6 (‘strongly agree’). By summing up EWB and RWB a total amount of Religious/Spiritual Well-Being (RSWB) is calculated. Cronbach’s α was at least at .72 for all the sub-dimensions and .89 for RSWB [38].

#### Mood pathology

The *Brief Symptom Inventory-18* (BSI-18) [39] is a short version of the highly established Symptom-Checklist SCL-90-R. The amount of psychiatric burden (Somatization, Depression, and Anxiety) for the preceding seven days is assessed with 18 items (6 per scale). The BSI-18 employs a 5-point likert scale (0 = ‘absolutely not’ to 4 = ‘very strong’). The total for the 18 items generate the Global Severity Index (GSI). Cronbach’s α was at least at .79 for the sub-dimensions and .91 for the GSI [39].

#### Statistical analyses

Pearson`s correlation statistics were conducted in order to investigate the relationships between study variables. Hierarchical regression analyses were conducted to examine the influence of attachment and spirituality on the dimensions of mood pathology. To control for alpha inflation due to multiple comparisons alpha-level was set to *p* < .01.

## Results

### Participants

The mean age of the participants was 23 years (*SD* = 2.93). 363 (76%) were female. 261 (54%) were in a romantic relationship. Only a few participants (*n* = 15; 3%) had children. The nationality of most of the participants (*n* = 441; 92%) was Austrian, 40 (8%) had other nationalities. For highest completed education level, 352 (73%) had a high school diploma while 129 (27%) had a university degree.

### Hypothesis testing results

We conducted correlation analyses (see Table 1) followed by hierarchical regression analyses (see Table 2) to examine the influence of attachment and spirituality dimensions on mood pathology.

**Table 1:** Descriptive characteristics and intercorrelations of study variables.

Measures	α	Min.	Max.	*M*	*SD*	1	2	3	4	5	6	7	8	9
**BSI-18**														
**1. Anxiety**	.76	0	22	4.57	3.94	-								
**2. Depression**	.84	0	24	5.09	5.02	.53**	-							
**3. Somatization**	.72	0	19	3.15	3.53	.49**	.41**	-						
**4. GSI**	.87	0	64	12.80	10.11	.82**	.85**	.74**	-					
**ECR-RD**														
**5. AX**	.92	1	6.50	3.11	1.20	.31**	.46**	.22**	.43**	-				
**6. AV**	.93	1	6.33	2.45	1.05	.16**	.26**	.12**	.24**	.40**	-			
**MI-RSWB**														
**7. EWB**	.84	42	139	107.02	15.48	-.20**	-.42**	-.13**	-.33**	-.38**	-.37**	-		
**8. RWB**	.86	38	136	76.65	18.91	-.11	-.18**	-.08	-.16**	-.28**	-.14**	.52**	-	
**9. RSWB**	.90	89	267	183.67	29.99	-.17**	-.33**	-.12	-.27**	-.38**	-.28**	.84**	.90**	-

**p < .01

BSI-18 = Brief Symptom Inventory-18, GSI = Global Severity Index, ECR-RD = Experience in Close Relationships–Revised, AX = Anxious Attachment, AV = Avoidant Attachment, MI-RSWB = Multidimensional Inventory of Religious/Spiritual Well-Being, EWB = Existential Well-Being, RWB = Religious Well-Being, RSWB = Religious/Spiritual Well-Being.

**Table 2:** Hierarchical regression analyses predicting mood pathology.

		Anxiety			Depression			Somatization			GSI		
Step and predictor variable		*R*^*2*^	*ΔR*^*2*^	*β*	*R*^*2*^	*ΔR*^*2*^	*β*	*R*^*2*^	*ΔR*^*2*^	*β*	*R*^*2*^	*ΔR*^*2*^	*β*
**Step 1**		.01	.01		.00	.00		.03**	.03**		.02**	.02**	
	**Sex**			-.12			-.03			-.18**			-.12**
**Step 2**		.11**	.10**		.22**	.22**		.08**	.05**		.20**	.19**	
	**Sex**			-.11			-.01			-.18**			-.11**
	**AX**			.28**			.42**			.18**			.38**
	**AV**			.07			.10			.08			.10
**Step 3**		.12**	.01		.29**	.07**		.08**	.00		.23**	.03**	
	**Sex**			-.11			-.03			-.19**			-.12**
	**AX**			.25**			.35**			.16**			.33**
	**AV**			.04			.02			.06			.05
	**EWB**			-.11			-.33**			-.06			-.23**
	**RWB**			.03			.10			.01			.06

**p < .01

Sex: 1 = male, GSI = Global Severity Index (BSI-18), AX = Anxious Attachment (ECR-RD), AV = Avoidant Attachment (ECR-RD), EWB = Existential Well-Being (MI-RSWB), RWB = Religious Well-Being (MI-RSWB).

As expected, correlation analyses showed that higher levels of AX and AV were associated with a higher level of mood pathology (AX: *r* = .43, *p* < .01; AV: *r* = .24, *p* < .01) and a lower level of RSWB (AX: *r* = -.38, *p* < .01; AV: *r* = -.28, *p* < .01). More specifically, the strongest correlations were found for AX with Depression (*r* = .48, *p* < .01) and with EWB (*r* = -.38, *p* < .01) respectively. Lower RSWB was also associated with a higher level of mood pathology (*r* = -.27, *p* < .01). Here, the strongest correlation was found for EWB with Depression (*r* = -.42, *p* < .01).

In the hierarchical regression analyses, sex was entered as a control variable at Step 1 as we found higher levels of GSI in female compared to male participants (*M*_female_ = 13.51, *M*_male_ = 10.63; *F*_(1, 480)_ = 7.35, *p* < .01). AX and AV were entered at Step 2, RWB and EWB at Step 3. The hierarchical regression analyses, including all predictors and the control variable, accounted for 12% of the variance in Anxiety (*F*_(5, 475)_ = 12.69, *p* < .01), 29% of the variance in Depression (*F*_(5, 475)_ = 38.80, *p* < .01), 8% of the variance in Somatization (*F*_(5, 475)_ = 8.75, *p* < .01), and 23% of the variance in GSI (*F*_(5, 475)_ = 28.95, *p* < .01).

At Step 1, sex was negatively related to Somatization and GSI (Somatization: *β* = -.18, *p* < .01; GSI: β = -.12, *p* < .01) but unrelated to Anxiety and Depression. At Step 2 and Step 3, *β* for sex changed only slightly.

At Step 2, AX was positively related to all three dimensions of mood pathology and GSI (Anxiety: *β* = .28, *p* < .01; Depression: *β* = .42, *p* < .01; Somatization: *β* = .18, *p* < .01; GSI: *β* = .38, *p* < .01). AV was not related to mood pathology.

At Step 3, AX was still positively related to all dimensions of mood pathology although β were smaller than at Step 2. AV was not related to mood pathology. RWB also had no significant relation to mood pathology while EWB was negatively related to Depression (*β* = -.33, *p* < .01) and GSI (*β* = -.23, *p* < .01) but unrelated to Anxiety and Somatization. Accordingly, only the models on Depression and GSI showed a significant increase in R^2^ between Step 2 and Step 3 (Depression: *ΔR*^*2*^ = .07, *p* < .01; GSI: *ΔR*^*2*^ = .03, *p* < .01).

## Discussion

In this study we sought to investigate the relationship between attachment and mood pathology as being possibly influenced by spirituality in a group of young adults. In accordance with previous research [40], we found secure attachment as to be associated with a higher amount of Religious/Spiritual Well-being (RSWB) while insecure attachment and lower RSWB were associated with higher levels of mood pathology [41]. This was especially pronounced for Depression. When attachment style and RSWB are considered together, only Anxious Attachment (AX), but not Avoidant Attachment (AV), positively predicted all three dimensions of mood pathology while Existential Well-Being (EWB), but not Religious Well-Being (RWB), was an additional negative predictor only for Depression. Results further suggest a mediating function of EWB on the positive relationship between insecure attachment and mood pathology.

### Attachment related coping strategies

The strong influence of AX on mood pathology might be explained through different coping strategies related to AX and AV: Impaired emotion regulation [42] and amplified distress due to hyperactivating strategies [7] likely contribute to our finding that AX predicts mood pathology with the strongest relation to depression. On the other hand, deactivating strategies reflect a compromise between a lack of visible negative emotions and high levels of unconscious distress [15]. Our finding that AV does not predict mood pathology independent of AX underlines that in these ‘segregated’ mental systems [42] distressing material, including personal weaknesses, is excluded from the stream of consciousness. As these deactivation strategies are often unreliable [43], it is possible that AV does increase psychopathology but that avoidant individuals are less likely to acknowledge their symptoms and to seek help. Accordingly, hyperactivating strategies seem to mediate AX and depressive symptoms while evidence for deactivating strategies as a mediator is mixed [13] and should be further explored in future research.

### Correspondence and compensation pathway

Our findings furthermore suggest that EWB, but not RWB, predicts mood pathology independent of attachment. This underlines previous research showing that RWB and EWB represent two independent concepts with different patterns of association to personality domains [44]. The correlation between RWB and mood pathology might simply occur because the relationship between believers and God often meet the established criteria of attachment relationships and therefore confers the same sorts of psychological advantages [20]. However, while the relation between higher RSWB and more secure attachment supports the correspondence pathway, EWB also seems to bear a protective effect regarding mood pathology that is independent of attachment style. This in turn supports the compensation pathway. EWB includes immanent hope, forgiveness, and experience of sense and meaning, dimensions that do not require an attachment relationship with a higher power. Increased EWB may develop through the ‘secure base’ [5], formed by a secure attachment to primary caregivers and/or a higher power. This ‘secure base’ liberates the individual to perceive self and others as separate with wishes and needs of their own so that both autonomy and connectedness are developed [1]. Interestingly, heightened autonomy, spirituality and self-acceptance also seem to have a positive effect on remission from disordered eating behavior [45].

### Methodological Considerations

The presented results can only be interpreted in light of potential limitations. Accordingly, as the current research focused on young adults with Roman Catholic upbringing, results may not be generalizable to individuals from other religious backgrounds. However, the detailed evaluation of religious and spiritual well-being can be considered as a strength of our study. Simply measuring whether someone belongs to a religious denomination might have led to inconclusive results because it is likely not the same as having spiritual or religious practices and beliefs [46].

Considering attachment, we only assessed current attachment style, while past experiences seem to be stronger predictors of religiosity, even though current attachment is largely based on these experiences [47]. However, reliance on self-report measures that access primarily conscious attitudes and behaviors may be a limitation in the assessment of both current attachment and past experiences since AV is often not captured as it leads individuals to normalize and report positively about themselves [48]. To better understand the implications of different attachment styles it may also be advisable to include constructs such as moral concern or mentalization in future studies. It has yet to be clarified whether deficits in this ability of self-projection that allows us to predict what others will think, feel or do in a certain situation [49] constrain the belief in God [50] or are unrelated to religious and spiritual beliefs after moral concern has been controlled for [51].

Finally, within-person variability in mood pathology over time and non-ignorable non-response (e.g., if depressed individuals were unwilling to participate because of their depression) may lead to an overrepresentation of healthy individuals in our sample. Further research in a clinical setting is therefore warranted.

### Conclusion

Our study contributes to the fields of developmental and clinical psychology by emphasizing the vital roles of attachment and existential well-being in young adult’s mental health. It can therefore inform future studies on the importance of spiritual development across the span of adult life [52]. A higher existential well-being seems to be able to especially compensate for the negative impact of insecure attachment on mental health. Therefore, our findings have clear implications for prevention and treatment given that the relative importance of attachment styles and religious/spiritual well-being might help tailor interventions to better address the specific needs of young adults.
